# Rapid Response Teams’ Initiative: Critical Role and Impact on National and Eastern Mediterranean Regional Emergency Management Capacity Building

**DOI:** 10.2196/14349

**Published:** 2019-10-16

**Authors:** Rawan Araj, Ali Odatallah, Jawad Mofleh, Sahar Samy, Nissaf Ben Alaya, Sultan Alqasrawi

**Affiliations:** 1 Global Health Development and the Eastern Mediterranean Public Health Network Amman Jordan; 2 Egypt Ministry of Health Cairo Egypt; 3 Ministry of Health, Tunisia Tunis Tunisia; 4 Directorate of Communicable Diseases, Jordan Ministry of Health Amman Jordan

**Keywords:** response teams, emergency, management, capacity building, training

## Abstract

Rapid response teams (RRTs) are essential to contain the harmful effects of emergency situations and to coordinate actions in the fragile environment of the Eastern Mediterranean region (EMR). The Global Health Development and the Eastern Mediterranean Public Health Network (EMPHNET) implemented RRTs to fill the human resources gap and to enable the member states to build their capacity in rapid assessment and response to public health events to reduce human suffering. To build the capacity of the member states in the field of rapid response and to build a strong team of rapid response specialists at the regional level, EMPHNET implemented this initiative at two levels. The first was a basic regional RRT course (July 2012). It was an introductory course for the selected candidates to provide insight and to enhance the knowledge and skills needed to be part of an RRT. The training included 32 participants from nine EMR countries. The course was designed to allow the facilitators and selection committee to select 15 to 20 potential candidates for the advanced RRT course. The second was the advanced RRT course (September 2010 to October 2012) for training the trainers and preparing the RRTs for deployment. A series of RRT training workshops were held, with more than 650 health staff from 12 countries trained. In all workshops that were conducted during 2016-2017, the trainees showed significant improvement in their knowledge and skills.

## Outbreaks and Conflict

In 2017, more than 76 million people were directly or indirectly affected by political conflict, war, displacement, environmental threats, famine, and natural disasters across the Eastern Mediterranean region (EMR) [1]. These conflicts and threats have resulted in many infectious disease outbreaks because of overcrowded living conditions, limited access to safe water and sanitation, and limited access to health care services [2]. Such threats necessitate effective rapid response mechanisms, training, equipment, and access to information [3].

With the 2005 revisions of the International Health Regulations (IHR, 2005), epidemic alerts and responses are critical to ensuring global health security [4]. Consequently, rapid response teams (RRTs) are essential to contain the harmful effects of emergency situations and to coordinate actions in the fragile environments, such as that of the EMR. The Centers for Disease Control and Prevention and the World Health Organization (WHO) recommended RRT training to rapidly contain outbreaks, such as avian influenza outbreaks in Southeastern Asia and the Middle East. According to the WHO’s assessments, the EMR needs better coordination of qualified individuals and increased capacity to detect and respond to disease outbreaks in a timely manner. Such individuals can serve in RRTs.

## Rapid Response Teams Initiative in the Eastern Mediterranean Region

RRT is a multistage collaborative project initiated by the Global Health Development (GHD) and the Eastern Mediterranean Public Health Network (EMPHNET) to fill the human resources gap and to enable the member states to build their capacity in rapid assessment and response to public health events to reduce human suffering. The GHD/EMPHNET had established the RRT initiative in 2012 to support the countries of EMR to effectively respond to public health events, including emerging infectious diseases and other biological threats in line with IHR [4]. The RRT initiative aims to increase the alert, investigation, and response capacity of the countries’ public health workforce, particularly graduates of the Field Epidemiology Training Program and the Public Health Empowerment Program, from both human and animal health sectors. Moreover, it aims to develop internal expertise in emergency preparedness and response operation, which will contribute to building stronger preparedness and response capacity of the countries and contribute to health security at the national, regional, and global levels. In addition, RRTs aimed to improve disease surveillance, outbreak investigation, and public health responses to disasters and disease outbreaks in the EMR, utilize skilled human resources in the EMR by developing a roster of well-trained RRTs at the national and subnational levels, and increase linkages among the public health institutions in the EMR for cross-border investigations of diseases/outbreaks.

## Strategy of Capacity Building in Rapid Response Teams

To build the capacity of the member states in the field of rapid response and to build strong teams of multisectoral rapid response specialists, EMPHNET first supported an intercountry training workshop, followed by the provision of technical support to some countries to replicate the training and build their rapid response framework (RRT at national and subnational levels). The intercountry workshop was a basic RRT course (July 1 to July 5, 2012). It was an introductory course for the selected candidates to provide insight and enhance the knowledge, competencies, and skills needed to be part of an RRT. The training included 32 participants from 9 EMR countries. The course was a good opportunity for the GHD/EMPHNET to identify potential future regional RRT members, as well as 15 to 20 potential candidates for the RRT course replication in respective countries. This activity was followed by an advanced RRT course (September 16 to October 4, 2012), where potential future regional RRT members were targeted. The advanced course was conducted over a 3-week duration and trained 19 professionals demonstrating commitment, knowledge, and enthusiasm to perform fieldwork under diverse situations.

The RRT trainings tackled issues related to the composition of an outbreak team, intersectoral coordination, IHR, surveillance systems, outbreak investigation and control measures, risk assessment, risk communication, role of the laboratory in outbreak investigation, biosafety and biosecurity, data management and report writing, and soft skills, among other topics.

After the regional RRT network was developed and fully functional, the trained RRT members continued training the national RRTs. The number and composition of these teams depended on each country’s needs. The EMPHNET aimed to provide a response platform at the national and subnational levels. The teams included epidemiologists, laboratory technicians, and other qualified public health officers from the ministries of health and individuals from the ministries of agriculture, including those who were working in animal husbandry, veterinary medicine, and disaster management. The strategy in training these national RRTs was to enable subregional trainings that mobilize a larger number of people and maximize resources where they are most needed.

## Achievements

The EMPHNET has successfully conducted both introductory and advanced courses on RRT training in the EMR. The introductory course included 32 participants from 9 countries: Afghanistan, Egypt, Iraq, Jordan, Lebanon, Saudi Arabia, Pakistan, Sudan, and Yemen. EMPHNET conducted a pretest, 3 quizzes, and a posttest, with a class average of 58.7%, 80.3%, and 89.6%, respectively. Comparison of pretests and posttests demonstrated an average class improvement of 30.9%. The participants’ grading of the workshop demonstrated a high level of satisfaction based on the postworkshop satisfaction survey. More than 95% of the participants strongly agreed that the workshop met their expectations.

On the basis of the results of the basic RRT training course, 19 participants scored at least 72% on the posttest and, therefore, they were qualified for the more extensive and advanced training. The 19 individuals included 4 from Yemen; 3 each from Afghanistan and Iraq; 2 each from Lebanon, Pakistan, Sudan, and Jordan; and 1 from Saudi Arabia. The evaluation of the 3-week advanced course also demonstrated a high level of satisfaction among the participants. The goal of these courses was to develop a highly qualified public health RRT to be ready to respond to disease outbreaks in the region and arrange subsequent RRT trainings at the national and provincial/governorate levels in their respective countries. This worked toward achieving the ultimate goal of having qualified regional and national RRTs in the EMR.

A series of RRT training workshops were held, where more than 650 health staff from 12 countries were trained. In all 10 workshops that were conducted during 2017-2018, the trainees showed significant improvement in their knowledge and skills, evaluated using pretest and posttest assessments (Table 1).

**Table 1:** The average pretest and posttest scores for participants who participated in 10 rapid response teams training workshops during 2016-2018.

Country	Date	Trainees, n	Pretest score (%), mean (SD)	Posttest score (%), mean (SD)
Egypt	April 24 to April 28, 2016	24	60 (13)	68 (7)
Egypt	July 29 to August 2, 2018	30	56 (11)	84 (6)
Egypt	September 23 to September 27, 2018	30	16 (9)	93 (3)
Egypt	April 14 to April 19, 2018	24	62 (10)	86 (12)
Egypt	July 8 to July 12, 2018	30	77 (11)	95 (3)
Tunisia	May 1 to May 11, 2018	73	60 (6)	69 (7)
Tunisia	November 6 to November 10, 2017	35	62 (10)	77 (6)
Sudan	June 24 to June 28, 2018	40	53 (8)	74 (14)
Morocco	October 30 to November 3, 2017	32	62 (10)	77 (6)
Jordan	March 12 to March 16, 2017	24	61 (15)	76 (8)

## Conclusions

Through the RRT initiative, the GHD/EMPHNET has developed a highly qualified public health RRT, which can roll out subsequent RRT trainings at the national and provincial/governorate levels in the EMR countries and contribute to responding to unexpected events in the region. For this developed capacity to be sustained and functional, the RRTs will need to be continuously engaged with the activities of public health stakeholders by participating in different efforts such as outbreak investigations or even simulation exercises.

## Abbreviations

EMPHNET: Eastern Mediterranean Public Health Network
EMR: Eastern Mediterranean region
GHD: Global Health Development
IHR: International Health Regulations
RRT: rapid response team
WHO: World Health Organization
